# Global DNA hypomethylation of colorectal tumours detected in tissue and liquid biopsies may be related to decreased methyl-donor content

**DOI:** 10.1186/s12885-022-09659-1

**Published:** 2022-06-02

**Authors:** Krisztina A. Szigeti, Alexandra Kalmár, Orsolya Galamb, Gábor Valcz, Barbara K. Barták, Zsófia B. Nagy, Sára Zsigrai, Ildikó Felletár, Árpád V. Patai, Tamás Micsik, Márton Papp, Eszter Márkus, Zsolt Tulassay, Peter Igaz, István Takács, Béla Molnár

**Affiliations:** 1 Department of Internal Medicine and Oncology, Faculty of Medicine, Semmelweis University, 1083 Budapest, Hungary; 2 Molecular Medicine Research Group, Eötvös Loránd Research Network, 1083 Budapest, Hungary; 3 Department of Surgery, Transplantation and Gastroenterology, Semmelweis University, 1082 Budapest, Hungary; 4 Interdisciplinary Gastroenterology (IGA) Working Group, Semmelweis University, 1082 Budapest, Hungary; 5 1st Department of Pathology and Experimental Cancer Research, Semmelweis University, 1085 Budapest, Hungary; 6 Centre for Bioinformatics, University of Veterinary Medicine Budapest, 1078 Budapest, Hungary; 7 Department of Anaesthesia and Intensive Care, Pest County Flor Ferenc Hospital, 2143 Kistarcsa, Hungary; 8 Department of Internal Medicine and Hematology, Faculty of Medicine, Semmelweis University, 1088 Budapest, Hungary; 9 Department of Endocrinology, Faculty of Medicine, Semmelweis University, 1083 Budapest, Hungary

**Keywords:** Colorectal cancer, Epigenetics, DNA methylation, Liquid biopsy, Folic acid, S-adenosylmethionine

## Abstract

**Background:**

Hypomethylation of long interspersed nuclear element 1 (LINE-1) is characteristic of various cancer types, including colorectal cancer (CRC). Malfunction of several factors or alteration of methyl-donor molecules’ (folic acid and S-adenosylmethionine) availability can contribute to DNA methylation changes. Detection of epigenetic alterations in liquid biopsies can assist in the early recognition of CRC. Following the investigations of a Hungarian colon tissue sample set, our goal was to examine the LINE-1 methylation of blood samples along the colorectal adenoma-carcinoma sequence and in inflammatory bowel disease. Moreover, we aimed to explore the possible underlying mechanisms of global DNA hypomethylation formation on a multi-level aspect.

**Methods:**

LINE-1 methylation of colon tissue (*n* = 183) and plasma (*n* = 48) samples of healthy controls and patients with colorectal tumours were examined with bisulfite pyrosequencing. To investigate mRNA expression, microarray analysis results were reanalysed in silico (*n* = 60)*.* Immunohistochemistry staining was used to validate DNA methyltransferases (*DNMTs)* and folate receptor beta *(FOLR2)* expression along with the determination of methyl-donor molecules’ in situ level (*n* = 40).

**Results:**

Significantly decreased LINE-1 methylation level was observed in line with cancer progression both in tissue (adenoma: 72.7 ± 4.8%, and CRC: 69.7 ± 7.6% vs. normal: 77.5 ± 1.7%, *p* ≤ 0.01) and liquid biopsies (adenoma: 80.0 ± 1.7%, and CRC: 79.8 ± 1.3% vs. normal: 82.0 ± 2.0%, *p* ≤ 0.01). However, no significant changes were recognized in inflammatory bowel disease cases. According to in silico analysis of microarray data, altered mRNA levels of several DNA methylation-related enzymes were detected in tumours vs. healthy biopsies, namely one-carbon metabolism-related genes—which met our analysing criteria—showed upregulation, while *FOLR2* was downregulated. Using immunohistochemistry, *DNMTs,* and *FOLR2* expression were confirmed. Moreover, significantly diminished folic acid and S-adenosylmethionine levels were observed in parallel with decreasing 5-methylcytosine staining in tumours compared to normal adjacent to tumour tissues (*p* ≤ 0.05).

**Conclusion:**

Our results suggest that LINE-1 hypomethylation may have a distinguishing value in precancerous stages compared to healthy samples in liquid biopsies. Furthermore, the reduction of global DNA methylation level could be linked to reduced methyl-donor availability with the contribution of decreased *FOLR2* expression.

**Supplementary Information:**

The online version contains supplementary material available at 10.1186/s12885-022-09659-1.

## Background

Colorectal cancer (CRC) is the third most commonly diagnosed cancer type and possesses the second highest mortality worldwide, as reported by the 2020 GLOBOCAN survey [1].

The vast majority of sporadic CRCs develop from healthy epithelium through benign colorectal adenoma (AD) stages into CRCs [2]. CRC progression can also be associated with the presence of long-lasting inflammatory bowel diseases (IBD), including ulcerative colitis and Crohn’s disease [3, 4]. The prevalence of IBD is increasing continuously worldwide; thus, it is necessary to investigate the molecular background of this disease more thoroughly [5].

In most CRC cases, the main molecular driving force in tumour development is chromosomal instability, where extensive DNA hypomethylation can be detected as an early molecular event, contributing to the formation of chromosomal instability [6–8]. As a consequence of global DNA hypomethylation, mobile genetic elements such as long interspersed nuclear element 1 (LINE-1) can be released from the inhibition induced by DNA methylation [9–11]. As copies of LINE-1 retrotransposon compose about 17% of the human genome and their methylation level correlates well with genome-wide DNA methylation [12, 13], LINE-1 bisulfite pyrosequencing is utilised for global DNA methylation estimation. Minimally invasive diagnostic tests, such as the detection of cancer-specific molecular markers in cell-free DNA (cfDNA) fraction by blood sampling, can contribute to the early recognition of cancer formation and thereby can increase the survival rate [14–22]. Due to this fact and the prognostic value of LINE-1 methylation status in various cancer types [23], it is important to explore the possible clinical potential of LINE-1 hypomethylation detection.

DNA methyltransferases, demethylases, chromatin remodelling enzymes, transcription factors, non-coding RNAs, and methyl-donor molecules are necessary for maintaining a cell-specific DNA methylation pattern [24]. Malfunctions related to any of these factors can contribute to abnormal global DNA hypomethylation [24]. In methylation processes, the methyl group originates from the methyl-donor S-adenosylmethionine (SAM) molecule, which is produced by the methionine-cycle as part of one-carbon metabolism [25]. One-carbon metabolism consists of several closely connected pathways centered around folic acid (FA), which is responsible for carrying single carbon units, like methyl groups [25].

Several studies have focused on the influence of methyl-donor molecule intake on CRC development risk revealing inverse correlations [26–30]. However, results regarding the effect of methyl-donors on global DNA- and LINE-1 methylation are controversial. No LINE-1 methylation alteration was detected, ensuing folate supplementation in the case of normal colon tissue [31, 32], but a slight positive association was observed between serum folate level and global DNA methylation according to Pufulete et al. [33]. Furthermore, a trend of association was noticed between LINE-1 methylation level and folate intake in studies about patients possessing colorectal tumours with DNA hypomethylation [34, 35]. It is also important to note that folate supply could have a different effect on pre-established neoplasms than on normal tissue, as it can contribute to the progression of the former and the maintenance of the latter [36]. As global DNA hypomethylation is closely connected to chromosomal instability along with CRC development, and folic acid intake may have a protective effect before neoplastic transformation, the investigation of the possible underlying mechanisms—including aberrant methyl-donor availability—is crucial.

The present study aims to comprehensively evaluate LINE-1 methylation on a Hungarian sample cohort along the colorectal adenoma-carcinoma sequence using LINE-1 bisulfite-sequencing as a basis for our further examinations. Moreover, we involved liquid biopsy samples to analyse the potential diagnostic value of LINE-1 hypomethylation, including the investigation of patients with IBD due to its high incidence rate and risk of CRC development. Finally, we aimed to explore the possible molecular background behind the emergence of global DNA hypomethylation during colorectal tumour progression on a multi-level aspect. Our purpose was to combine microarray analysis and immunohistochemistry staining to discover possible connections between DNA methylation-related enzymes’ mRNA expression profile, methyl-donor (FA and SAM) content, and global DNA hypomethylation.

## Material and methods

### Sample collection

A total of 183 fresh frozen (FFT), 40 formalin-fixed paraffin-embedded tissue (FFPET) biopsies, and 48 blood samples taken in K3EDTA Vacuette tubes (Greiner Bio-One Gmbh) were collected at the Department of Internal Medicine and Hematology and the 1^st^ Department of Pathology and Experimental Cancer Research, Semmelweis University, Budapest, Hungary.

The FFT sample cohort contained 45 healthy (N), 23 normal adjacent to colorectal adenoma tissue (AD-NAT), 25 normal adjacent to colorectal carcinoma tissue (CRC-NAT), 37 AD, 38 CRC, and 15 IBD biopsy samples. AD samples included different histological subtypes, namely tubular (TA) and tubulovillous (TVA) biopsies, while the CRC group contained specimens with multiple Astler-Coller modified Dukes’ A-B (early) and Astler-Coller modified Dukes’ C-D (late) stages. NAT biopsies were taken 10–12 cm distance from the lesions. FFPET biopsies included 20 AD and 20 CRC sections containing transitional zones. Moreover, plasma samples of 10 healthy, 14 AD, 13 CRC, and 11 IBD patients were also involved in this study. Exclusion criteria were acute health problems, cancerous diseases apart from CRC, radiotherapy, and chemotherapy. The clinicopathological and demographic data of the investigated patients are summarized in Additional file 1 (Supplemental material Table 1, 2, 3).

The study has been approved by the local ethics committee (Regional and Institutional Committee of Science and Research Ethics; TUKEB Nr: 14,383–2/2017/EKU), and all applied methods were performed in accordance with the relevant guidelines and regulations. Written informed consent was obtained from all the patients prior to sample collection.

### DNA isolation from fresh frozen tissue and plasma samples

Genomic DNA was isolated from FFT biopsies with High Pure PCR Template Preparation Kit (Roche) according to the manufacturer’s instruction with overnight proteinase K digestion.

Plasma fractions were separated from whole blood samples by two centrifugation steps (1350 rcf, 12 min). CfDNA isolation was carried out from 3.5 ml plasma samples with Quick-cfDNA™ Serum & Plasma Kit (Zymo Research) according to the manufacturer's recommendation. DNA samples were stored at -20 °C until further examinations. Concentration and purity (OD260/280, OD230/280) of genomic DNA were measured by NanoDrop ND-1000 Spectrophotometer (Thermo Fisher Scientific), while cfDNA quantification was performed with Qubit 1.0 fluorometer using Qubit dsDNA HS Assay Kit (Thermo Fisher Scientific).

### LINE-1 bisulfite pyrosequencing

After DNA isolation, 500 ng DNA of each tissue sample and approximately 20 ng cfDNA of each plasma sample were bisulfite-converted using EZ DNA Methylation-Direct Kit (Zymo Research) according to the manufacturer's protocol. A 146 basepair-long region of LINE-1 sequence was amplified by bisulfite-specific PCR using Pyromark Q24 CpG LINE-1 Kit (Qiagen) on Mastercycler EP Gradient S PCR machine (Eppendorf, Hamburg, Germany) with the following thermocycling program: PCR activation for 15 min on 90 °C; 45 cycles of 30 s denaturation on 94 °C, 30 s annealing on 50 °C and 30 s extension on 72 °C; 10 min final extension on 72 °C. The specificity of PCR was verified by gel electrophoresis using 2% agarose gel. Amplicons were prepared and washed on PyroMark Q24 Vacuum Workstation (Qiagen) and pyrosequenced on Pyromark Q24 (Qiagen) instrument using Pyromark Q24 CpG LINE-1 Kit (Qiagen) and PyroMark Gold Q24 Reagents (Qiagen). Methylation of three cytosine-guanine dinucleotides (positions 318, 321, and 328 of LINE-1, GenBank accession number: X58075) was calculated as the percentage of cytosine nucleotides relative to the sum of cytosine and thymine nucleotides by Pyromark Q24 software v2.0.6. The average methylation level of the three cytosine-guanine dinucleotides was assessed as the LINE-1 methylation status of each sample. All pyrosequencing and analysis steps were performed according to the manufacturer's description.

Statistical significance (*p* ≤ 0.05) and receiver operating characteristic (ROC) curve analysis were calculated using Prism8 software (GraphPad). In multiple comparison tests, data with non-normal distribution were analysed by Kruskal–Wallis and Dunn's multiple comparison tests, while in the case of normal distribution, ANOVA followed by Brown Forsythe and Welch ANOVA tests were applied. In pairwise comparisons, paired t-test or Wilcoxon matched-pairs signed rank tests were performed depending on normal or non-normal data distribution, respectively.

### In silico gene expression analysis

Human Transcriptome Array (HTA) 2.0 RNA microarray (Affymetrix) was performed by Kalmár et al. according to the manufacturer’s recommendations to detect the mRNA expression profile of 20 colorectal normal, 20 AD, and 20 CRC samples [37]. The dataset can be found on Gene Expression Omnibus (GEO) data repository (GEO ID GSE100179) [37]. Processed data were reanalysed with Transcriptome Analysis Console 4.0 software (TAC 4.0, Affymetrix). Criteria of our in silico analysis were the following: adjusted *p*-value ≤ 0.05; fold change (FC) ≥|1.4|.

### Immunohistochemistry

Immunohistochemistry staining was applied on 5 µm sections obtained from 20 AD and 20 CRC FFPET samples. After deparaffinization, heat-induced epitope retrieval was performed. Tissue sections were treated with 3% hydrogen peroxide solution for 30 min. Nonspecific binding sites were blocked with 1% BSA solution for 60 min at room temperature. According to the manufacturers’ protocol, the following primary antibodies were used: anti-DNMT1 (AB19905, Abcam), anti-DNMT3a (ab16704, Abcam), anti-DNMT3b (ab122932, Abnova), and anti-FOLR2 (ab228643, Abcam, 1:500), which is specific to folate receptor beta (FR-beta) encoded by *FOLR2*. In the case of FA, SAM, and 5-methylcytosine (5mC) staining, sections were firstly treated with 0.1% Tween-TBS for 10 min after 30 min of 3% hydrogen peroxide incubation. Antigen retrieval was performed with 2 N HCl solution for 15 min at room temperature. The pH was normalized using 100 mM TBS (pH 8.3). Nonspecific binding sites were blocked with 1% BSA for 30 min. Primary antibodies were applied according to the manufacturers’ recommendation (anti-folic acid (PAB0092, Abnova, 1:500), anti-S-adenosylmethionine (Clone 118–18, 6942–25, BioVision, 1:200), anti-5-methylcytosine (ab214727, Abcam, 1:500).

Secondary antibodies were utilised for 1 h at room temperature: anti-rabbit antibody (BA1054, Boster), anti-rat antibody (18–4818-82, Invitrogen), and anti-mouse antibody (62–6520, Invitrogen). The visualisation was performed using DAB Chromogen (K3468, DAKO) with HISTOLS-DAB Substrate (30,014. K, Histopathology Ltd.), and nuclei were stained with hematoxylin.

Slides were digitised with Pannoramic Confocal instrument and were analysed using Pannoramic Viewer digital microscope (v: 1.15.3, 3DHISTECH). The intensity was classified from 0 to + 3, where 0 means negative, while + 1 is a weak, + 2 is an intermediate, and + 3 is a strong immunohistochemical signal. To evaluate the staining, modified Quick-score (Q-score) method was applied (Formula: Q-score = IxP, P: percentage of the positive cells, I: staining intensity of these cells, maximum value: 300 (3 × 100) [38, 39]). Statistical significance (*p* ≤ 0.05) was assessed by paired t-test, or Wilcoxon matched-pairs signed rank test depending on normal or non-normal data distribution, respectively (Prism8 software, GraphPad).

## Results

### LINE-1 methylation in tissue samples and plasma fraction

**Fig. 1:**
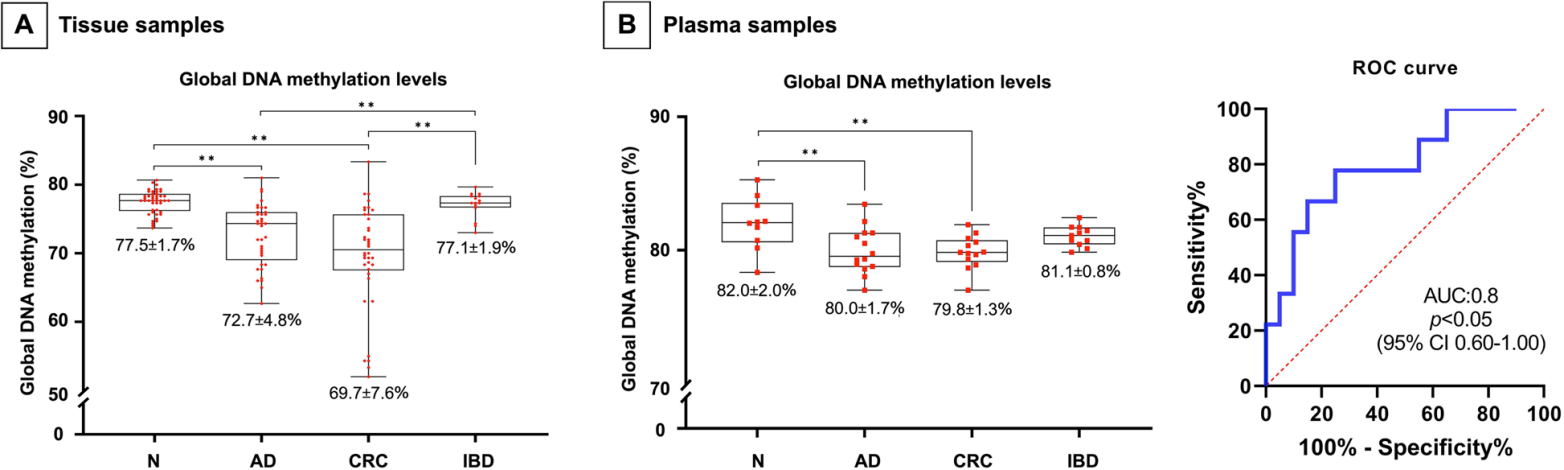
LINE-1 methylation of tissue, LINE-1 methylation, and receiver operating characteristic analysis of plasma specimens. **A** Significantly decreased LINE-1 methylation was observed in tumours compared to healthy and IBD tissue biopsies (**p ≤ 0.01). There were no significant methylation level alterations in IBD tissue samples in comparison with N biopsies. **B** Significant decrease of LINE-1 methylation was noticed in AD and CRC vs. N blood samples (**p ≤ 0.01). IBD specimens did not show significantly altered methylation level compared to healthy liquid biopsies (left). AD samples were separated from healthy controls with 66.7% sensitivity and 90.0% specificity (threshold 80.0%) (right). N: healthy, AD: colorectal adenoma, CRC: colorectal carcinoma, IBD: inflammatory bowel disease, ROC: receiver operating characteristic nalysis

Significant decrease of LINE-1 methylation was found in AD (72.7 ± 4.8%) and in CRC (69.7 ± 7.6%) samples compared to N (77.5 ± 1.7%) colon and IBD (77.1 ± 1.9%) tissue specimens according to LINE-1 bisulfite sequencing (*p* ≤ 0.01). However, there was no significant methylation alteration in IBD biopsies compared to N (Fig. 1/A).

AD-NAT and CRC-NAT specimens (76.0 ± 2.1%, 76.4 ± 2.0%, respectively) showed significantly reduced methylation levels compared to the samples of healthy individuals (77.5 ± 1.7%) (*p* ≤ 0.05) (Additional file 2/A). Furthermore, the LINE-1 methylation levels of AD (72.8 ± 4.4%) and CRC (71.2 ± 6.8%) tissue samples decreased significantly compared to their matched NAT pairs (76.0 ± 2.1%, 76.4 ± 2.0%, respectively) (*p* ≤ 0.05) (Additional file 2/B).

Significantly lower LINE-1 methylation was found in TA (73.8 ± 4.1%) and TVA (69.8 ± 4.5%) in comparison with N samples (77.5 ± 1.7%) (*p* ≤ 0.05), and a decreasing trend was observed in TVA (69.8 ± 4.5%) vs. TA (73.8 ± 4.1%) (Additional file 2/C, left). LINE-1 methylation levels of both early (71.5 ± 4.9%) and late (68.5 ± 8.7%) carcinomas were significantly reduced compared to N specimens (*p* ≤ 0.05) (Additional file 2/C, right). A decline was also noticed in late cancer stages (68.5 ± 8.7%) in comparison with early carcinoma (71.5 ± 4.9%) biopsies.

According to our observations, AD (80.0 ± 1.7%) and CRC (79.8 ± 1.3%) plasma specimens showed significantly decreased methylation status compared to N (82.0 ± 2.0%, *p* ≤ 0.01), while no significant alteration was observed in IBD (81.1 ± 0.8%) in comparison with N samples (Fig. 1/B, left). Receiver operating characteristic (ROC) curve analysis was applied in AD liquid biopsy samples to investigate the potential of LINE-1 methylation as a biomarker that already indicates the precancerous AD stages. At the cut-off value 80.0%, LINE-1 methylation percentage distinguished AD from N with 66.7% sensitivity and 90.0% specificity (AUC: 0.8, *p* < 0.05, 95% CI 0.6–1.0) (Fig. 1/B, right).

### In silico gene expression analysis of genes related to DNA methylation

GeneChip™ HTA 2.0 results of our research group were reanalysed in silico to investigate the mRNA expression profile of folate receptors, one-carbon cycle-, and DNA methylation-related enzymes [37].

**Fig. 2:**
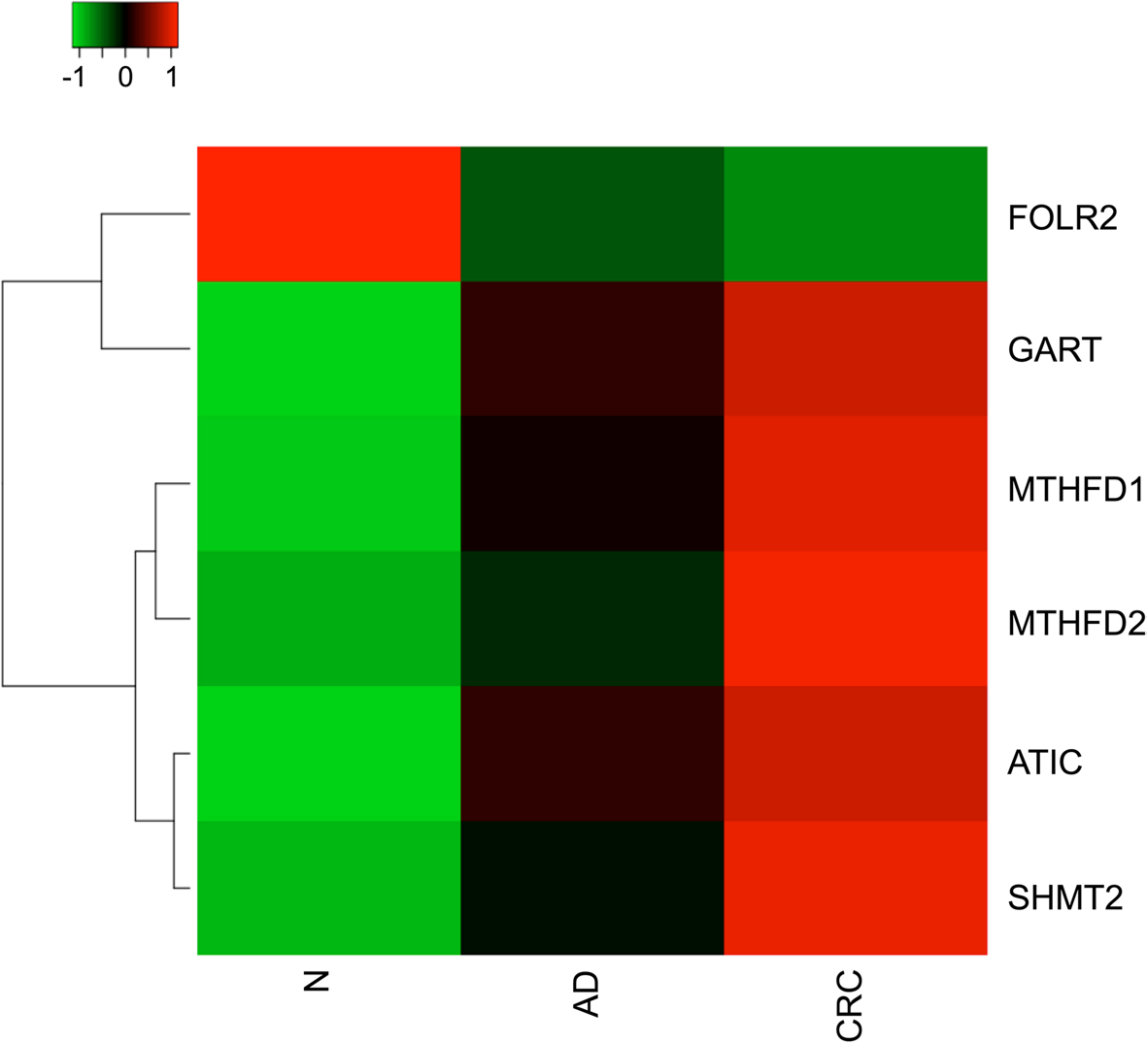
Significant mRNA expression alterations of folate receptors and genes involved in one-carbon cycle. On the heatmap, distinct colours are coupled to different expression intensity values: green - low, black - intermediate, red - high intensity. Each row represents different genes, and each column indicates the investigated sample types. Only candidates owning significant expression changes (*p* ≤ 0.05) and fold change value greater or equal than |1.4| in both AD and CRC tissue samples compared to N were presented on the heatmap. One-carbon metabolism-related enzymes showed significantly elevated mRNA expression in AD and CRC samples compared to N. *FOLR2* had a significantly lower transcript level in tumorous samples vs. healthy controls (*p* ≤ 0.05). N: healthy, AD: colorectal adenoma, CRC: colorectal carcinoma

Only candidates showing significant expression changes (*p* ≤ 0.05) in both AD and CRC tissue samples compared to N were illustrated in Fig. 2. Five genes involved in one-carbon metabolism (*GART, MTHFD1, MTHFD2, ATIC,* and *SHMT2*) were found to be overexpressed in tumorous samples in comparison with healthy controls, while *FOLR2* owned significantly reduced gene expression alterations with -1.45 and -1.53 FC values in AD vs. N and CRC vs. N comparisons (*p* ≤ 0.05), respectively (Fig. 2.).

*DNMT1* showed significant mRNA expression level elevation with 1.51 FC value in CRC vs. N comparison. *APOBEC1* also possessed increased transcription with 1.88 FC value in AD samples compared to N specimens (*p* ≤ 0.05). *APOBEC3B* had significantly decreased transcript level (FC = -1.49) in AD vs. healthy biopsies (*p* ≤ 0.05). mRNA level alterations of *DNMT1*, *APOBEC1*, *APOBEC3B* are represented on Additional file 3.

### Validation of DNMTs and FOLR2 GeneChip™ Human Transcriptome Array 2.0 results

#### Gene expression results of *DNMTs* and *FOLR2* were validated with immunohistochemistry staining

DNMT1 labelling showed significantly elevated intensity in CRC epithelial cells (*p* ≤ 0.05), while no expression changes were detected in stromal cells of CRC compared to NAT. No significant alterations could be observed in DNMT3A and DNMT3B immunostaining between the analysed areas (Additional file 4).

Significantly lower (*p* ≤ 0.01) *FOLR2*-encoded FR-beta expression was detected in epithelial and stromal cells of AD and epithelial cells of CRC specimens compared to their NAT areas (Additional file 5). In CRC stromal cells, a moderate reduction of staining intensity was noticed in comparison with NAT regions.

### Analyses of methyl-donor content in colon tissue

**Fig. 3:**
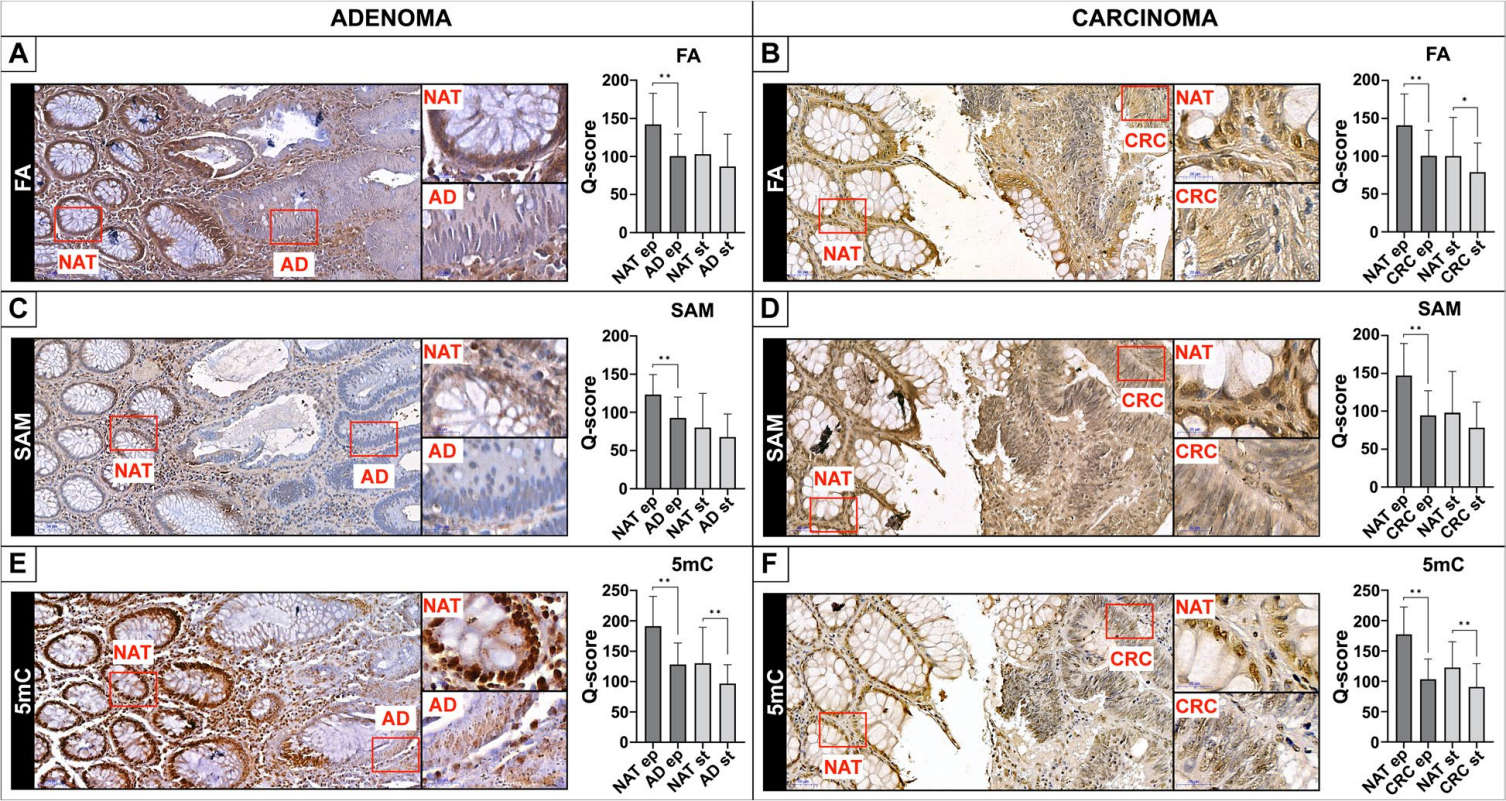
Immunohistochemistry staining of methyl-donor molecules and 5mC in AD and CRC tissue samples. Significantly lower staining intensity of FA (**A**, **B**), SAM (**C**, **D**), and 5mC (**E**, **F**) was observed in epithelial cells of both AD and CRC regions compared to NAT areas (**p ≤ 0.01). Slightly reducing trend was noticed in stromal cells except for 5mC labelling in AD (**E**), and CRC (**F**) (**p ≤ 0.01), along with FA staining in CRC specimens (**B**) (*p ≤ 0.05) also possessing significantly lower immunostaining. Q-score method was applied to evaluate the staining intensity of immunohistochemistry. Scale bars on the left: 50 µm, on the right: 20 µm. FA: folic acid, SAM: S-adenosylmethionine, 5mC: 5-methylcytosine, NAT: normal adjacent to tumour tissue, AD: colorectal adenoma, CRC: colorectal carcinoma, ep: epithelial cells, st: stromal cell, Q-score: Quick-score method

Immunohistochemistry staining was applied to investigate in situ methyl-donor (FA and SAM) content and examine its relation to the 5mC level. Significantly reduced intensity of FA (Fig. 3/A, B), SAM (Fig. 3/C, D), and 5mC (Fig. 3/E, F) was observed in epithelial cells of both AD and CRC vs. NAT areas (*p* ≤ 0.01). Slightly diminished labelling was noticed in stromal cells except for 5mC staining in AD (Fig. 3/E) (*p* ≤ 0.01), and CRC (Fig. 3/F) (*p* ≤ 0.01), along with FA labelling in CRC sections (Fig. 3/B) (*p* ≤ 0.05), where also significantly lower intensity was detected.

## Discussion

Significant LINE-1 hypomethylation has been known in several cancers compared to healthy tissue specimens [40]. In the present study, we focused specifically on tissue samples collected from Hungarian individuals, revealing significant LINE-1 hypomethylation in both AD and CRC biopsies compared to N, paired AD-NAT, and CRC-NAT tissues. Moreover, our measurements of AD-NAT, CRC-NAT, and N colonic biopsies showed significantly lower LINE-1 methylation in NAT samples vs. healthy controls, which also agreed with the recent scientific literature [8, 41, 42].

Significant LINE-1 hypomethylation was noticed in TA and TVA samples compared to N, and tendentiously lower methylation levels were found in TVA specimens compared to TA biopsies. In contrast with the latter result, no LINE-1 methylation alteration was detected in TVA vs. TA comparison, according to the observations of Jiang et al. [43]. Our result can be explained by the fact that ADs with more villous features have a higher risk of cancerous transformation [44], and global DNA methylation is continuously decreasing with CRC progression [45].

Significant reduction of LINE-1 methylation was detected in the samples of patients with early and late CRC stages compared to N specimens, while moderately diminished methylation level was observed within the CRC group (Dukes C-D vs. Dukes A-B). This is consistent with the fact that global DNA methylation level reduces during CRC development [45, 46].

The molecular examination of IBD is crucial due to its increasing incidence and its relation to the risk of CRC development [5, 47, 48]. Glória et al. found global DNA hypomethylation in rectal mucosa of ulcerative colitis patients compared to those who had negative colonoscopy [49]. As a novelty of the present study, no significant LINE-1 methylation changes were observed in the IBD vs. N comparison. Nevertheless, AD and CRC tissue samples showed significantly lower LINE-1 methylation levels than IBD. Similar to our observations, significantly declined LINE-1 methylation level was identified in gastric cancer compared to paired NAT samples, but no significant alteration was noticed between healthy gastric, NAT, and chronic gastritis tissue specimens [50].

The research of Nagai et al. was the first to describe LINE-1 hypomethylation as a possible diagnostic biomarker for early CRC detection using plasma samples [51]. As a novelty of our study, significant LINE-1 hypomethylation was also found in AD besides CRC samples compared to healthy controls in cfDNA fraction. Similar to our findings in inflamed tissue samples, no significant methylation alteration was detected in IBD vs. N plasma samples. Based on these results and the literature data mentioned above, LINE-1 hypomethylation can be considered a characteristic feature of tumour formation rather than inflammation, which can be advantageous in the development of a cancer-specific screening procedure based on LINE-1 hypomethylation. Since reduced LINE-1 methylation is characteristic in various cancer types, it may not serve as a specific diagnostic marker for CRC identification, but it can be useful in a personalized, minimally invasive monitoring method to observe AD formation, CRC progression, or remission [2]. The low number of investigated plasma samples is the limitation of this study; however, our results may expand the opportunities for monitoring colorectal tumour development with a more comfortable method for patients.

The phenomenon of LINE-1 hypomethylation is widely examined, but its development has not been fully understood yet. Thus, the expression alterations of DNA methylation-related enzymes and the in situ level of methyl-donor molecules were examined. Genes that met our investigating criteria during microarray analysis are discussed below. Upregulation of DNA demethylase enzymes can result in DNA hypomethylation [52]. Hence, the transcript level of TETs and APOBECs encoding genes were analysed [53–56], from which only *APOBEC1* and *APOBEC3B* had significant alterations. mRNA level of *APOBEC1* increased, and *APOBEC3B* diminished significantly in benign compared to normal specimens. As a higher transcript level of a catalytically inactive, shorter *APOBEC1* splice-variant has been observed in colorectal cancer before [57, 58], the elevated mRNA expression level also detected in our study may not have an effect on the global DNA methylation level. Regarding *APOBEC3B*, declined mRNA expression was noticed in CRC vs. N samples by Burns et al. as well [59]. In normal cells, APOBEC3 enzymes can inhibit the retrotransposition of LINE-1 sequences in a manner not coupled to DNA methylation [60, 61]. Consequently, the reduced level of *APOBEC3B* in colorectal cancer may contribute to the accumulation of LINE-1 copies not through lessened DNA demethylase activity but through decreased restriction of retrotransposons’ mRNA.

SAM producing methionine-cycle is part of the one-carbon metabolism [25]; therefore, the associated genes were also analysed in this study. Candidates of one-carbon cycle with mRNA expression alterations that met our criteria (*SHMT2, MTHFD1, MTHFD2, ATIC,* and *GART*) showed significant upregulation in AD and CRC vs. N cases. Overexpression of one-carbon metabolism-related enzymes is observed in many cancer types, including colon cancer, which can be explained by the elevated nucleotide demand of the highly proliferating cells [62].

FR-beta and DNMT enzymes are key factors of the DNA methylation process, as FR-beta provides a unidirectional transport of folate to the cells, while DNMTs are the main catalysts of DNA methylation [63, 64]. Hence, in our study, these enzymes were selected for validation with immunohistochemistry staining. Elevated *DNMT1* and no significantly altered *DNMT3a*, *-3b* expressions were verified by HTA 2.0 microarray analysis and immunohistochemistry. DNMT1 is responsible for the maintenance of global DNA methylation, while DNMT3a and 3b are liable for local, promoter-specific methylation patterns [65, 66]. Increased *DNMT1* expression can be related to a regulatory feedback mechanism, as DNA hypomethylation and elevated *DNMT1* mRNA levels were detected in response to inhibited DNA methylation [67–69].

Regarding *FOLR2,* we found reduced mRNA expression in polypoid and cancerous lesions compared to healthy tissues. Immunohistochemistry staining also confirmed decreased FR-beta expression in AD and CRC vs. NAT regions. Similar to our findings, weak staining intensity of tumorous cells was noticed by de Boer et al. [70]. The downregulation of *FOLR2* may result in decreased FA and SAM levels, which can impact DNA methylation pattern [24].

Immunohistochemistry staining was applied to examine the in situ tissue presence of methyl-donor molecules. Evaluation of in situ methyl-donor content in colorectal tissue with immunolabelling technique in parallel with 5mC staining was one of our novelties. FA and SAM labelling showed decreased intensity in adenomatous and cancerous areas compared to NAT regions in line with 5mC staining, which also reduced along the normal-adenoma-carcinoma sequence. Similarly, the study published by Alonso-Aperte et al. revealed decreased folate status in colonic neoplastic mucosa compared to normal and also reduced S-adenosylmethinone/S-adenosylhomocysteine ratio, which means diminished methylation potential [71]. Our observations about decreased SAM levels in tumorous areas can be explained by the fact that folate provides single carbon moieties for one-carbon metabolism, so the lower amount of this molecule can lead to decreased SAM level and lessened global DNA methylation [72, 73]. Even though recent findings regarding the effect of folate deficiency on DNA methylation and cancer progression are controversial [74], our results support the fact that low methyl-donor content may contribute to the formation of global DNA hypomethylation and CRC development.

## Conclusion

In summary, we observed significant LINE-1 hypomethylation along the colorectal adenoma-to-carcinoma continuum in both tissue and peripheral blood specimens in a Hungarian colon sample cohort. Plasma specimens are emphasised since intraindividual monitoring of LINE-1 hypomethylation may serve as an approach to provide assistance in the observation of AD formation, CRC progression, or remission. Additionally, LINE-1 methylation did not show a significant alteration in IBD. Hence, it might offer a higher screening value.

Our results on the gene expression changes of methylation- and one-carbon metabolism-related enzymes could not explain the decrease of global DNA methylation level; however, they may have complex regulatory mechanisms in the background. We presume that the diminished expression of *FOLR2* in colorectal tumours can contribute to the declined methyl-donor availability, which may lead to the process of global DNA hypomethylation.

## Supplementary Information

**Additional file 1.** Summary of LINE-1 methylation levels, clinicopathological and demographic data of the investigated patients

**Additional file 2.** LINE-1 methylation in NAT, tumour and paired NAT specimens, AD subtypes, and CRC stages. Significant LINE-1 methylation reduction was noticed in the NAT vs. N comparison (**A**) and in tumour specimens compared to their paired NAT tissue biopsies (**B**) (*p≤0.05). In TA, TVA vs. N comparison, significantly lower LINE-1 methylation was found (*p≤0.05), and a decreasing trend was observed in TVA compared to TA biopsies (C, left). Also, significant LINE-1 hypomethylation was detected in both early (Astler-Coller modified Dukes’ A-B) and late (Astler-Coller modified Dukes’ C-D) carcinomas compared to N specimens (C, right) (*p≤0.05). N: healthy, AD NAT: normal adjacent to adenoma tissue, CRC NAT: normal adjacent to carcinoma tissue, AD: colorectal adenoma, CRC: colorectal carcinoma, TA: tubular adenoma, TVA: tubulovillous adenoma

**Additional file 3.** mRNA expression alterations of *DNMT1*, *APOBEC1*, *APOBEC3B* genes in tumorous compared to normal samples. On the heatmap, distinct colours are coupled to different expression intensity values: green - low, black - intermediate, red - high intensity. Each row represents different genes, and each column indicates the investigated sample types. *DNMT1* mRNA level increased significantly only in CRC vs. N comparison, while *APOBEC1* significantly elevated, and *APOBEC3B* significantly decreased only in AD samples compared to healthy controls (*p*≤0.05).  

**Additional file 4. **Expression changes of DNMT enzymes in CRC tissue sections. A) Significant increase of DNMT1 labelling in the epithelial cells (*p≤0.05) and no altered expression in stromal cells were detected in the cancerous area compared to NAT. There were no significant changes in DNMT3a (B) and DNMT3b (C) staining in CRC vs. NAT comparison. Expression differences were illustrated with the Q-score method (below). Scale bars on the top: 1000µm, on the bottom: 20µm. NAT: normal adjacent to tumour tissue, CRC: colorectal carcinoma, ep: epithelial cells, st: stromal cells, Q-score: Quick-score method

**Additional file 5. **Immunohistochemistry of FR-beta encoded by FOLR2 in AD and CRC samples. Significant reduction of FR-beta intensity was found in epithelial and stromal cells in the AD (**A**) and CRC (**B**) vs. NAT comparison (**p≤0.01) except for CRC stromal cells, where a slight decrease was observed (**B**). Histological changes are represented with the Q-score method on the right. Scale bars on the left: 50µm, on the right: 20µm. NAT: normal adjacent to tumour tissue, AD: colorectal adenoma, CRC: colorectal carcinoma, ep: epithelial cells, st: stromal cells, Q score: Quick-score method 

## Data Availability

The microarray dataset analysed during the current study are available in the Gene Expression Omnibus (GEO) data repository (accession number: GSE100179).

## Abbreviations

5mC: 5-methylcytosine
AD: Adenoma
APOBEC1: Apolipoprotein B mRNA editing enzyme catalytic subunit 1
APOBEC3B: Apolipoprotein B mRNA editing enzyme catalytic subunit 3B
ATIC: 5-aminoimidazole-4-carboxamide ribonucleotide formyltransferase/IMP cyclohydrolase
CfDNA: Cell-free DNA
CRC: Colorectal cancer
DNMT1: DNA methyltransferase 1
DNMT3a: DNA methyltransferase 3a
DNMT3b: DNA methyltransferase 3b
FA: Folic acid
FC: Fold change
FFT: Fresh frozen tissue
FFPET: Formalin-fixed paraffin-embedded tissue
FOLR2: Folate receptor beta
FR-beta: Folate receptor beta
GART: Phosphoribosylglycinamide formyltransferas, phosphoribosylglycinamide synthetase, phosphoribosylaminoimidazole synthetasee
GEO: Gene Expression Omnibus
HTA: Human Transcriptome Array
IBD: Inflammatory bowel disease
LINE-1: Long interspersed nuclear element 1
MTHFD1: Methylenetetrahydrofolate dehydrogenase, cyclohydrolase and formyltetrahydrofolate synthetase 1
MTHFD2: Methylenetetrahydrofolate dehydrogenase (NADP + Dependent) 2, methenyltetrahydrofolate cyclohydrolase
NAT: Normal adjacent to tumour tissue
Q-score: Quick-score method
ROC: Receiver operating characteristic
SAM: S-adenosylmethionine
SHMT2: Serine Hydroxymethyltransferase 2
TA: Tubular adenoma
TVA: Tubulovillous adenoma
