# Supplementary material for: Global DNA hypomethylation of colorectal tumours detected in tissue and liquid biopsies may be related to decreased methyl-donor content
Source: BMC Cancer. 2022 Jun 2;22:605. doi: 10.1186/s12885-022-09659-1 (PMC9164347; doi:10.1186/s12885-022-09659-1)
Supplement: Supplementary file 1 — Additional file 1. Summary of LINE-1 methylation levels, clinicopathological and demographic data of the investigated patients [file 12885_2022_9659_MOESM1_ESM.pdf]

Table 1. Clinicopathological and demographic data of healthy patients analysed in this study.

Variables		Tissue		Plasma	
		n	Methylation level (%) ± SD	n	Methylation level (%) ± SD
Age	≤65	38	77.5 ± 1.7	10	82.0 ± 2.0
	>65	7	77.4 ± 1.7	-	-
Sex	Female	30	77.2 ± 1.6	5	81.1 ± 1.9
	Male	15	78.0 ± 1.7	5	82.9 ± 1.8

Table 2. Clinicopathological and demographic data of patients with colorectal adenoma and carcinoma analysed in this study.

Colorectal adenoma		Tissue					Plasma	
		FFT Tumour		FF NAT		FFPET		
		n	Methylation level ± SD	n	Methylation level ± SD	n	n	Methylation level ± SD
**Age**	≤65	15	73.0 ± 4.4	8	75.4 ± 1.2	13	6	79.7 ± 1.2
	>65	22	72.4 ± 4.5	15	76.3 ± 2.4	7	8	80.1 ± 2.1
**Sex**	female	15	70.4 ± 4.2 *	12	76.3 ± 2.2	11	10	79.4 ± 1.4
	male	22	74.2 ± 4.0	11	75.6 ± 2.1	9	4	81.5 ± 1.8
**Histological type of AD**	TA	25	73.7 ± 4.1	16	75.8 ± 2.0	8	6	80.0 ± 1.4
	TVA	9	70.7 ± 3.9	5	76.7 ± 1.8	11	5	79.7 ± 2.4
	both	3	69.8 ± 6.2	2	-	-	2	-
	N/A	-	-	-	-	1	1	-
**Degree of the dysplasia in AD**	low grade	29	72.9 ± 4.4	21	75.8 ± 2.0	1	4	80.1 ± 1.2
	high grade	4	75.6 ± 3.5	1	-	2	0	-
	both	1	-	1	-	-	-	-
	N/A	3	68.8 ± 0.8	-	-	17	10	79.9 ± 2.0
**AD size**	<10 mm	16	75.0 ± 3.1 *	14	75.5 ± 2.2	8	3	78.9 ± 0.9
	≥10 mm	7	67.9 ± 3.7	4	76.9 ± 1.7	1	5	80.5 ± 2.4
	both	11	72.8 ± 4.4	5	76.6 ± 2.1	4	5	80.3 ± 1.4
	N/A	3	70.4 ± 3.6	-	-	7	1	-
**AD Number**	1	9	70.9 ± 4.0	5	75.7 ± 2.1	3	6	80.1 ± 2.4
	≥2	26	73.4 ± 4.6	18	76.1 ± 2.2	16	7	80.0 ± 1.3
	N/A	2	-	-	-	1	1	-
**Colorectal cancer**								
**Age**	≤65	10	71.0 ± 4.9	8	75.8 ± 2.4	10	5	80.0 ± 1.8
	>65	28	69.1 ± 8.4	17	76.4 ± 1.8	10	8	79.8 ± 0.9
**Sex**	female	13	70.5 ± 8.3	8	76.1 ± 1.9	10	7	79.7 ± 1.4
	male	25	69.2 ± 7.4	17	76.2 ± 2.1	10	6	80.0 ± 1.1
**CRC stage (Astler-**	early (A+B)	13	71.8 ± 5.1	9	75.8 ± 1.9	9	2	-

**Coller modified Dukes')**	late (C+D)	23	68.1 ± 8.5	15	76.1 ± 1.7	8	9	79.5 ± 1.3
	N/A	2	-	1	-	3	2	-
**Location**	colon	26	69.1 ± 7.8	16	76.1 ± 2.2	10	9	79.8 ± 0.8
	rectum	12	70.8 ± 7.4	9	76.3 ± 1.7	10	2	-
	N/A	-	-	-	-	-	2	-
**Lymph node metastasis**	0	13	72.0 ± 4.9	8	76.1 ± 1.8	9	1	-
	≥1	14	66.8 ± 9.6	14	76.2 ± 1.8	6	9	77.5 ± 4.2
	N/A	11	70.4 ± 6.7	3	76.8 ± 3.7	5	3	80.5 ± 1.2
**Distant metastasis**	0	16	71.8 ± 4.6	11	75.7 ± 1.8	16	1	-
	≥1	14	68.4 ± 10.0	10	76.4 ± 1.7	4	6	79.1 ± 1.3
	N/A	8	67.4 ± 7.2	4	77.1 ± 3.1	-	6	80.6 ± 0.9

Statistical significance is indicated with * ($p \leq 0.05$). AD: adenoma, NAT: normal adjacent to tumour tissue, TA: tubular adenoma, TVA: tubulovillous adenoma, CRC: colorectal carcinoma.

Table 3. Clinicopathological and demographic data of patients with IBD analysed in this study.

Variables		Tissue		Plasma	
		**n**	**Methylation level ± SD**	**n**	**Methylation level ± SD**
**Age**	≤65	14	77.1 ± 2.0	11	81.1 ± 0.8
	>65	1	-	-	-
**Sex**	female	7	76.3 ± 2.0	5	80.8 ± 0.8
	male	8	77.8 ± 1.6	6	81.2 ± 0.8
**Type of IBD**	Crohn's disease	-	-	5	81.0 ± 0.9
	Ulcerative colitis	13	77.9 ± 0.9	3	81.1 ± 0.9
	N/A	2	73.8 ± 0.7	2	-

IBD: inflammatory bowel disease
