# Supplementary figures and images for: Global DNA hypomethylation of colorectal tumours detected in tissue and liquid biopsies may be related to decreased methyl-donor content
Source: BMC Cancer. 2022 Jun 2;22:605. doi: 10.1186/s12885-022-09659-1 (PMC9164347; doi:10.1186/s12885-022-09659-1)

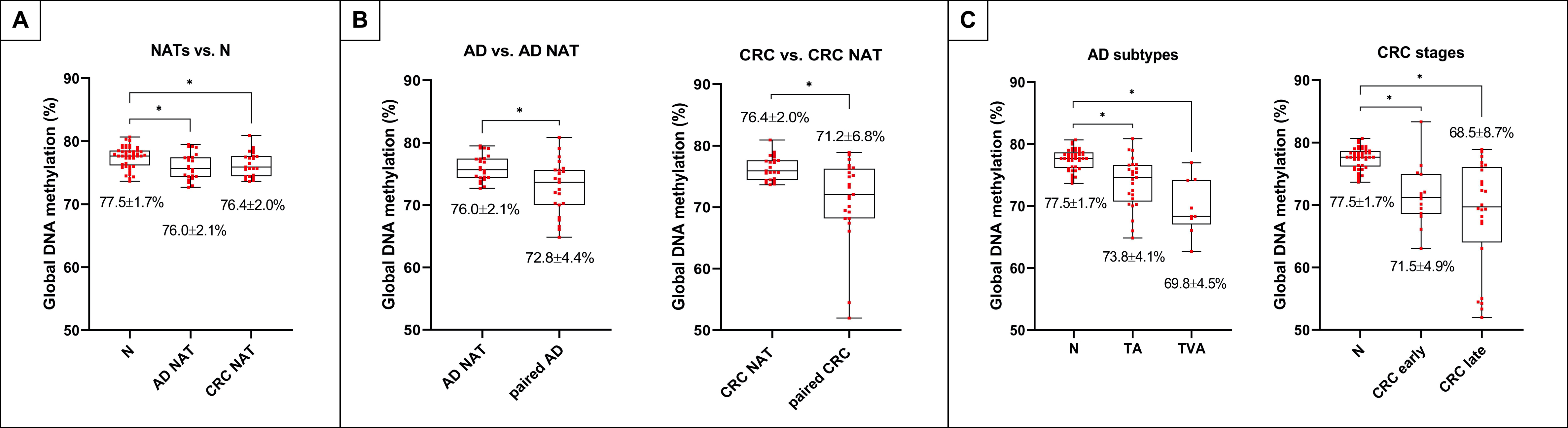

Supplement: Supplementary file 2 — Additional file 2. LINE-1 methylation in NAT, tumour and paired NAT specimens, AD subtypes, and CRC stages. Significant LINE-1 methylation reduction was noticed in the NAT vs. N comparison (A) and in tumour specimens compared to their paired NAT tissue biopsies (B) (*p≤0.05). In TA, TVA vs. N comparison, significantly lower LINE-1 methylation was found (*p≤0.05), and a decreasing trend was observed in TVA compared to TA biopsies (C, left). Also, significant LINE-1 hypomethylation was detected in both early (Astler-Coller modified Dukes’ A-B) and late (Astler-Coller modified Dukes’ C-D) carcinomas compared to N specimens (C, right) (*p≤0.05). N: healthy, AD NAT: normal adjacent to adenoma tissue, CRC NAT: normal adjacent to carcinoma tissue, AD: colorectal adenoma, CRC: colorectal carcinoma, TA: tubular adenoma, TVA: tubulovillous adenoma [file 12885_2022_9659_MOESM2_ESM.jpg]

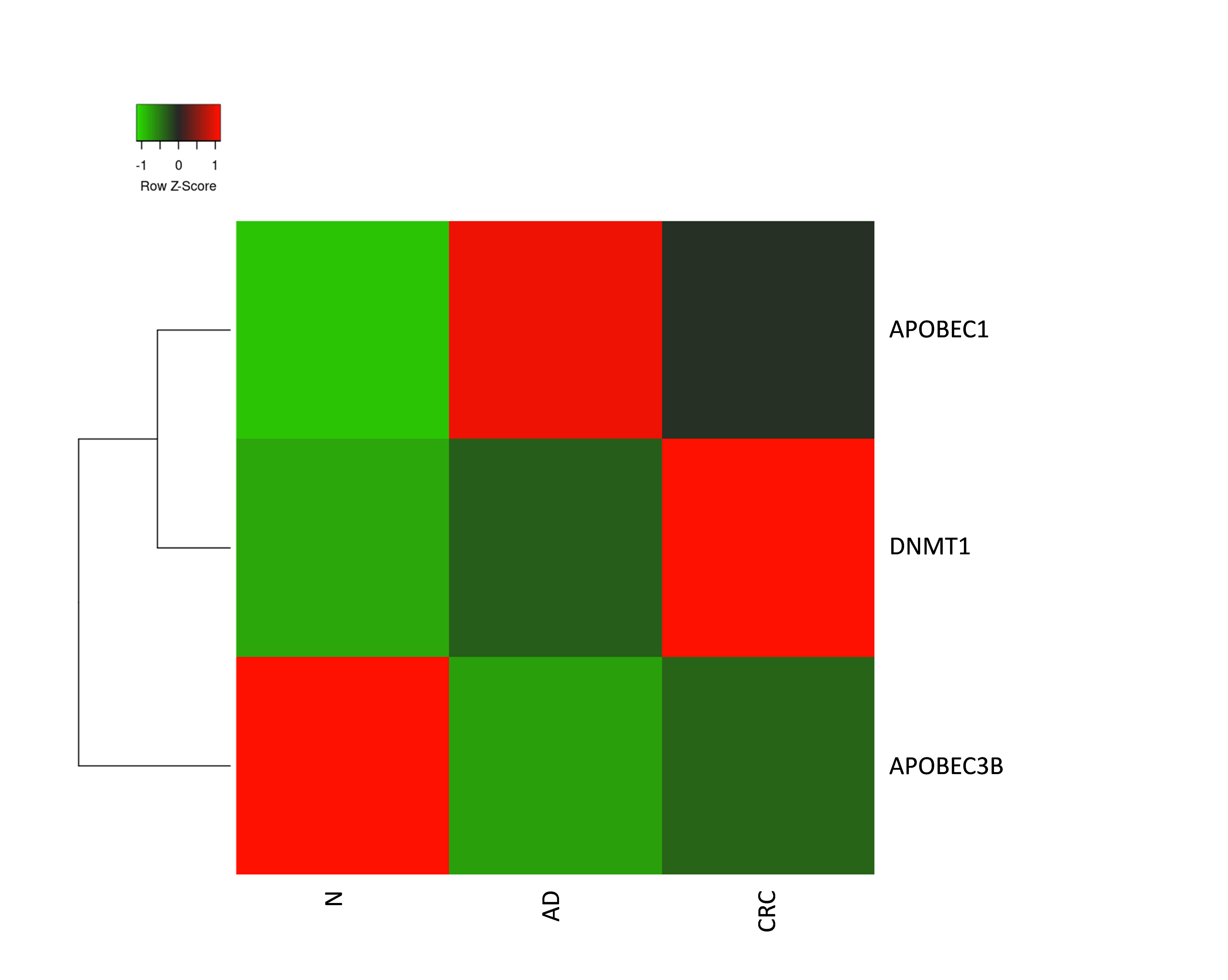

Supplement: Supplementary file 3 — Additional file 3. mRNA expression alterations of DNMT1, APOBEC1, APOBEC3B genes in tumorous compared to normal samples. On the heatmap, distinct colours are coupled to different expression intensity values: green - low, black - intermediate, red - high intensity. Each row represents different genes, and each column indicates the investigated sample types. DNMT1 mRNA level increased significantly only in CRC vs. N comparison, while APOBEC1 significantly elevated, and APOBEC3B significantly decreased only in AD samples compared to healthy controls (p≤0.05). [file 12885_2022_9659_MOESM3_ESM.jpg]

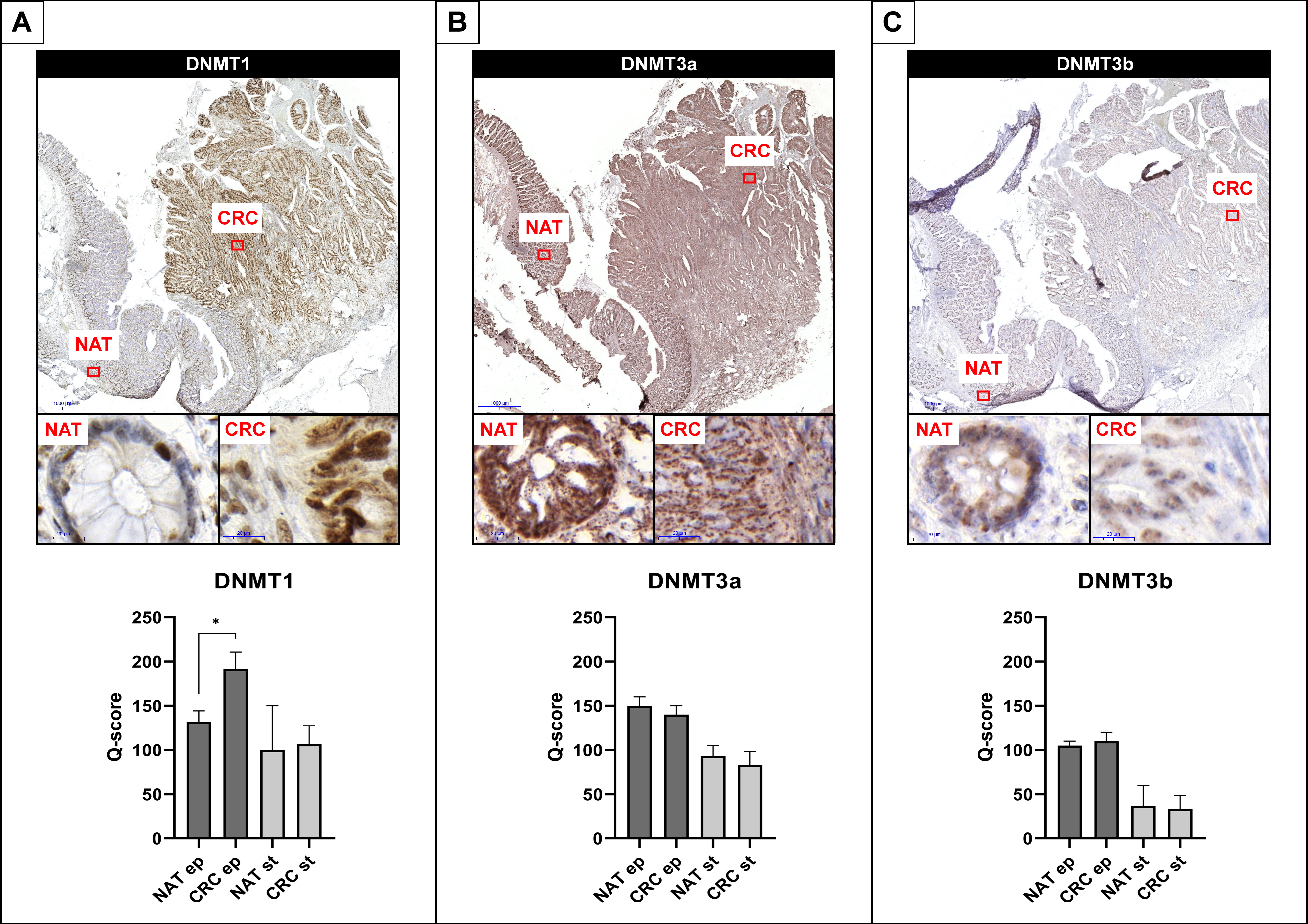

Supplement: Supplementary file 4 — Additional file 4. Expression changes of DNMT enzymes in CRC tissue sections. A) Significant increase of DNMT1 labelling in the epithelial cells (*p≤0.05) and no altered expression in stromal cells were detected in the cancerous area compared to NAT. There were no significant changes in DNMT3a (B) and DNMT3b (C) staining in CRC vs. NAT comparison. Expression differences were illustrated with the Q-score method (below). Scale bars on the top: 1000µm, on the bottom: 20µm. NAT: normal adjacent to tumour tissue, CRC: colorectal carcinoma, ep: epithelial cells, st: stromal cells, Q-score: Quick-score method [file 12885_2022_9659_MOESM4_ESM.jpg]

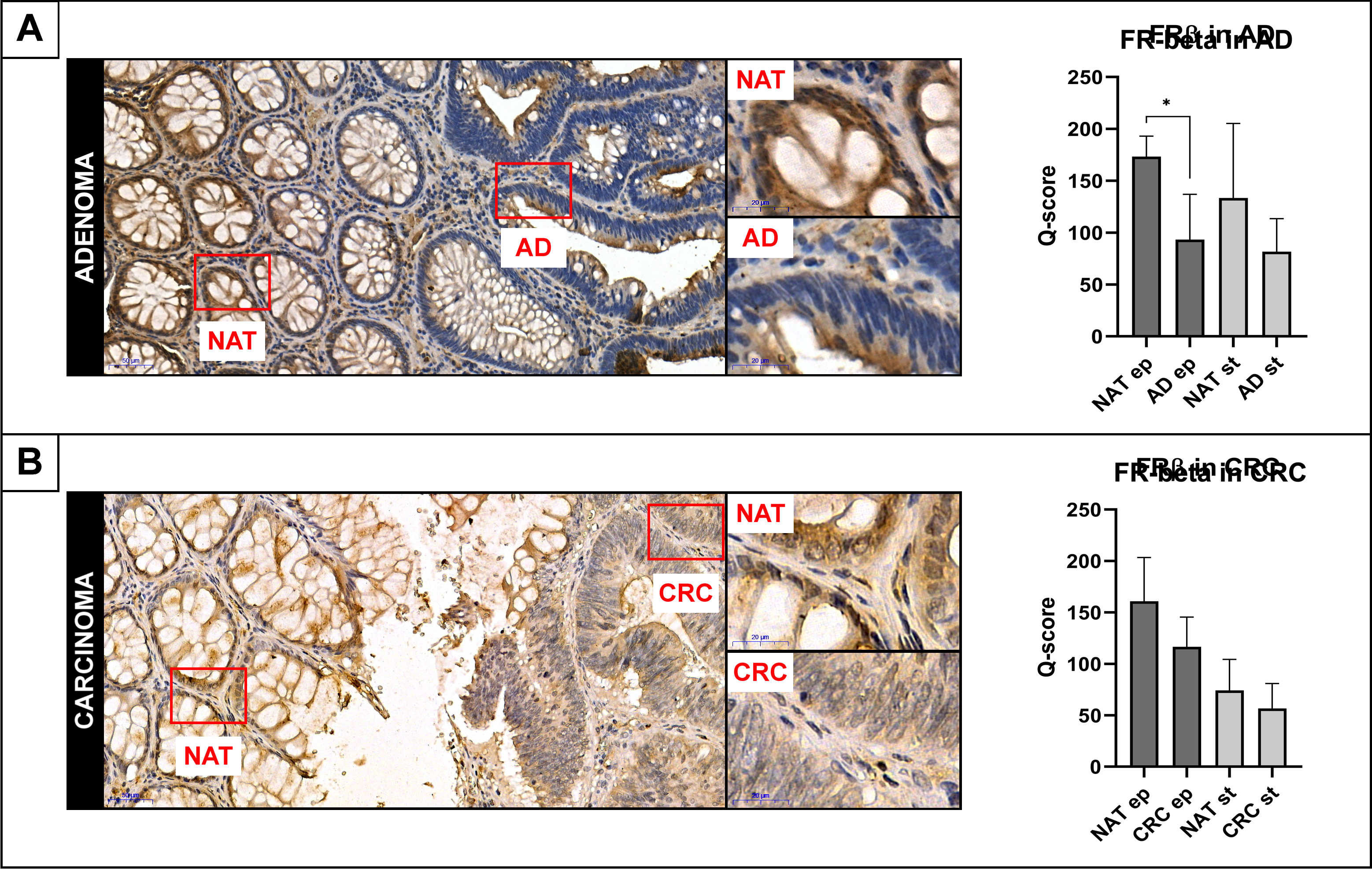

Supplement: Supplementary file 5 — Additional file 5. Immunohistochemistry of FR-beta encoded by FOLR2 in AD and CRC samples. Significant reduction of FR-beta intensity was found in epithelial and stromal cells in the AD (A) and CRC (B) vs. NAT comparison (**p≤0.01) except for CRC stromal cells, where a slight decrease was observed (B). Histological changes are represented with the Q-score method on the right. Scale bars on the left: 50µm, on the right: 20µm. NAT: normal adjacent to tumour tissue, AD: colorectal adenoma, CRC: colorectal carcinoma, ep: epithelial cells, st: stromal cells, Q score: Quick-score method [file 12885_2022_9659_MOESM5_ESM.jpg]
